# Complications after open fractures in children and adolescents - a single-center retrospective observational study

**DOI:** 10.1007/s00068-026-03109-4

**Published:** 2026-02-23

**Authors:** Alexis Brinkemper, Vanessa Marx, Thomas A. Schildhauer, Christiane Kruppa

**Affiliations:** Department of General and Trauma Surgery, BG-University Hospital Bergmannsheil Bochum, Ruhr-University Bochum, Bürkle-de-la-camp Platz 1, 44789 Bochum, Germany

**Keywords:** Open fractures, Children and adolescents, Complications, Infection, Antibiotics

## Abstract

**Purpose:**

Purpose of the study was to report the antibiotic treatment and complications after open fractures in children and adolescents at a level one trauma center.

**Methods:**

We retrospectively report data of 72 children and adolescents with 74 open fractures. The open fractures were evaluated according to severity, etiology, localization, concomitant injuries, therapeutic treatment, concomitant antibiotic treatment, complications and outcome.

**Results:**

We found a significant relationship between the occurrence of complications and the severity of the open fractures, the treatment method, the mechanism of injury and for the localization. A correlation between the occurrence of complications and the antibiotic treatment could not be shown.

**Conclusions:**

As the degree of injury increases, so does the likelihood of complications. There is also a correlation to treatment with external fixation, non-primary wound closure, traffic accidents and when the lower leg or foot is affected. However, the cause-and-effect relationship should always be considered.

## Background

Open fractures are a serious injury in which a bone fracture is accompanied by damage to the overlying soft tissues, including but not limited to the skin. The incidence ranges from 0.7 to 2% in children and adolescents [1, 2]. Higher-grade open fractures usually result from high-impact trauma such as traffic accidents or sports injuries, while punctiform impalements can also occur in low-energy injuries. The direct connection between the fracture and the external environment significantly increases the risk of contamination, which can lead to infections and increased morbidity [1]. Therefore, careful management, which in addition to fracture care also includes the prevention of infection, is crucial for the subsequent outcome [3]. While wound debridement and irrigation is widely suggested and adapted from the adult treatment, no consensus exists about duration of antibiotic treatment besides single shot antibiotics previously to the operation, especially in grade I° open fractures [4–6].

Despite the urgency and potential risks, open fractures in pediatric patients generally have a more favorable prognosis than in adults [1]. This is partly due to a greater potential for periosteal bone formation and thus faster bone healing in children [1]. The complication rates in children and adolescents are also described as lower than in adults [1, 4, 7].

The aim of this study is to retrospectively evaluate the complications after open fractures in children and adolescents at a single center and to analyze possible correlations with regard to the type of injury, the mechanism of injury, the type of surgical treatment, as well antibiotic administration.

## Methods

The present study was performed in accordance with the Declaration of Helsinki. Ethical permission for this study was obtained from the local ethics committee. This article is written following the STROBE recommendations for reports of cohort, case-control or cross-sectional studies. Children and adolescents, who were treated for open fractures of all body regions at our department of general and trauma surgery at a level one trauma center, between the years from 2006 to 2024 were identified. Inclusion criteria were age of 0 to 16 years, presence of an open fracture and complete radiological/clinical documents. Patients who died immediately due to the severity of the injury were excluded. We retrospectively reviewed patient’s charts and radiographs. Patient’s demographics were recorded. Open fractures were categorized according to Gustilo and Anderson [8]. The open fractures were evaluated according to severity, etiology, localization, concomitant injuries, therapeutic treatment, concomitant antibiotic treatment, complications and outcome. Follow-up data were included in the analysis from a minimum period of three months. In general, institutional antibiotic treatment consists of a single dose of a second-generation cephalosporin (usually Cefazolin), adapted to the patient’s weight, for all open fractures and closed fractures that require surgery. This is usually administered in the emergency department in cases of open fractures, or 30–15 min before operative treatment. For patients with open fractures of grades II–III, the attending surgeon makes an individual decision about the length of postoperative antibiotic treatment, depending on the characteristics of the wound, the degree of contamination, and the severity of the injury.

Depending on the characteristics of the injury, the attending surgeon made an individual decision about definitive or temporary fracture treatment. In cases of I° open fractures, definitive fracture treatment is usually performed after local debridement and primary wound closure in our institution. For more severe injuries, definitive fracture care and primary wound closure are only performed when reasonable. In all other cases, temporary fracture treatment and various types of temporary wound closure are used, depending on the individual situation. Primary wound closure usually involves suturing. For temporary closure, we use vacuum devices, Coldex wound dressings, dermotraction and sterile gauzes. In cases of delayed closure, depending on the characteristics of the soft tissue, a suture closure, skin mesh graft or local or free soft tissue/muscle flaps are used.

### Statistical analysis

Descriptive statistics were completed including percent, mean, range and standard deviation (SD). Evaluation of categorical variables was carried out using the Pearson’s Chi square test or Fisher’s exact test when there was expected cell counts < 5 in any cell. All the statistical analyses were carried out using SPSS Statistics 30.0.0.0 (IBM Corp., Armonk, NY: IBM Corp). *P* values ≤ 0.05 were determined as statistically significant.

## Results

### Patients and injuries

Our patient collective consisted of 72 children and adolescents with 74 open fractures, with an average age of 11.3 years (SD 3.7, range 2–16). During the investigation period, according to an estimate based on data from 2013 to 2025, there were approximately 1,615 fractures in children and adolescents in total at our clinic, which corresponds to an incidence of 4.6%. All evaluations in the further course refer to the number of open fracture cases (74). The average follow-up time was 11.4 months (SD 19.4, range 0-111) for all cases and 16.0 months (SD 21.9, range 3-111) for cases included in the follow-up analysis with at least three months follow-up. With 44.6%, I° open fractures were the most common, followed by 33.8% II° open and 21.6% III° open fractures. 75.0% of the III° open fractures were caused by traffic accidents. Demographic data, mechanism of injury, localization, severity classification and concomitant injuries are shown in Table 1.

In 25 (33.8%) cases, the primary diagnosis was made ex domo and in twelve (16.2%) of these cases, primary treatment was also carried out ex domo before a transfer to our hospital took place. The time between admission to hospital and surgical treatment was less than six hours in 64 cases (86.5%) and between six and twelve hours in six cases (8.1%). For three (4.1%) cases this information could not be reconstructed, one case (1.4%) was not surgically treated.

**Table 1:** Demographics, mechanism of injury, localization, severity and concomitant injuries of 72 children and adolescents with 74 open fractures. All percentages refer to the number of open fractures

	*n* (%) or mean (± SD, range)
Demography	
Male	48 (64.9)
Female	26 (35.1)
Age (years)	11.3 (3.7, 2–16)
Right side	30 (40.5)
Left side	44 (59.5)
Lower extremity	36 (48.6)
Upper extremity	38 (51.4)
Mechanism of injury	
Fall > 2 m	4 (5.4)
Fall < 2 m	35 (47.3)
Traffic accident	24 (32.4)
Impact trauma	5 (6.8)
Others	6 (8.1)
Localization	
Humerus	12 (16.2)
Forearm	23 (31.1)
Finger	3 (4.1)
Femur	5 (6.8)
Lower leg	22 (29.7)
Foot	9 (12.2)
Severity (Gustilo&Anderson)	
I°	33 (44.6)
II°	25 (33.8)
III° total	16 (21.6)
a	8 (10.8)
b	7 (9.5)
c	1 (1.4)
Concomitant injury (incl. multiple combination)	36 (48.6)
Polytrauma	13 (17.6)
At least 1 further closed fracture	15 (20.3)
Traumatic brain injury	3 (4.1)
Thoracic contusion / pneumothorax / lung contusion	6 (8.1)
Decollement/soft tissue injury at other locations	5 (6.8)
Vascular injury at other locations	2 (2.7)
Others	14 (18.9)

### Treatment

Osteosynthesis was performed in 48 cases (64.9%) using an immediate definitive procedure (Elastic stable intramedullary nailing (ESIN), K-wire, Plate). This was particularly the case for I° (29) and II° (14) open fractures. In contrast, initial treatment with an external fixator was required in 68.8% of the III° open fractures. In one patient (1.4%) with a I° open fracture of the digitus manus II the fracture was treated conservatively with a stack-splint after local wound debridement and primary wound closure outside the operating room. In total primary wound closure was achieved in 57 (77.0%), mainly I° and II° open fractures. In the III° open fractures, however, different procedures had to be used to achieve sufficient secondary soft tissue closure.

In 32 cases (43.2%), the antibiotic regimen consisted exclusively of preoperative single-shot antibiotics with a cephalosporin. In 21 cases (28.4%), antibiotics were also administered for up to seven days and in 14 cases (18.9%), antibiotic therapy was administered for more than seven days. Osteosynthesis, treatment of soft tissue defects and antibiotic therapy in relation to the severity of the fracture are shown in Table 2.

30 open fractures (40.5%) required more than one surgery as a staged procedure, e.g. due to a definitive osteosynthesis treatment after primary external fixation or due to a previous compartment splitting. However, this was also the case due to various complications. A list of the subsequent surgeries can be found in Table 3.

**Table 2:** Osteosynthetic procedures, treatment of soft tissue defects and antibiotic therapy in relation to the severity of the fracture

Total *n*	Grade I° *n* (%)	Grade II° *n* (%)	Grade III° *n* (%)	Total *n* (%)
	33	25	16	74
Primary fracture stabilization				
ESIN/K-wire/Plate	29 (87.9)	14 (56.0)	5 (31.3)	48 (64.9)
External fixator	3 (9.1)	9 (36.0)	11 (68.8)	23 (31.1)
Conservative outside the operating room	1 (3.0)	-	-	1 (1.4)
Surgical wound debridement and irrigation only	-	2 (8.0)	-	2 (2.7)
Treatment of soft tissue defects (incl. multiple combination)				
Primary wound closure	29 (87.9)	21 (84.0)	7 (43.8)	57 (77.0)
Secondary suture	1 (3.0)	3 (12.0)	4 (25.0)	8 (10.8)
Skin graft	1 (3.0)	2 (8.0)	3 (18.8)	6 (8.1)
Flap-plasty	-	-	3 (18.8)	3 (4.1)
Temporary procedures (dermotraction, VAC, alginates, compresses)	2 (6.1)	4 (16.0)	9 (56.3)	15 (20.3)
Conservative	1 (3.0)	-	-	1 (1.4)
Unknown	1 (3.0)	-	-	1 (1.4)
Antibiosis				
Single shot (SS) antibiosis only	17 (51.5)	9 (36.0)	6 (37.5)	32 (43.2)
SS + antibiosis ≤ 7 days	9 (27.3)	8 (32.0)	4 (25.1)	21 (28.4)
SS + antibiosis > 7 days	4 (12.1)	5 (20.0)	5 (31.3)	14 (18.9)
Local antibiosis (ointment)	1 (3.0)	-	1 (6.3)	2 (2.7)
Unknown	2 (6.1)	3 (12.0)	-	5 (6.8)

**Table 3:** List of subsequent surgeries for patients with more than one surgery to treat open fracture, *n* = 30

Sex	Age (years)	Localization	Severity (Gustilo&Anderson)	Primary fracture stabilization (intern/extern)	2nd surgery	3rd surgery	4th surgery	5th surgery and more
M	16	Lower leg	I°	Intern	Secondary suture, debridement	Secondary suture		
M	15	Foot	III° b	Extern	MR external fixator, definitive osteosynthesis	MR k-wire	MR	
M	10	Humerus	II°	Extern	MR external fixator, definitive osteosynthesis			
M	11	Forearm	I°	Intern	MR k-wire, definitive osteosynthesis			
F	10	Lower leg	III° b	Extern	Fasciotomy	Secondary suture	Secondary suture, soft tissue closure (flap)	5th Soft tissue closure (flap) 6th MR external fixator
F	13	Lower leg	III° b	Extern	MR external fixator, definitive osteosynthesis	Definitive osteosynthesis		
M	3	Lower leg	III° c	Extern	Debridement	Debridement	Soft tissue closure (flap)	5th Debridement 6th MR external fixator
M	10	Femur	III° a	Extern	Secondary suture	MR external fixator		
M	13	Lower leg	II°	Extern	MR external fixator, definitive osteosynthesis			
F	5	Lower leg	III° a	Extern	Fasciotomy	Secondary suture	Secondary suture	MR external fixator
M	8	Lower leg	III° b	Extern	Soft tissue closure (flap)	Soft tissue closure (flap)	MR external fixator	
M	15	Lower leg	I°	Extern	Fasciotomy	Secondary suture	MR external fixator, definitive osteosynthesis	
M	16	Foot	III° a	Extern	MR external fixator			
M	13	Lower leg	II°	Extern	MR external fixator, definitive osteosynthesis	Soft tissue closure (flap)		
M	10	Lower leg	II°	Extern	Fasciotomy	Secondary suture	MR external fixator	
M	12	Foot	II°	Intern	Amputation distal phalanx D1			
F	8	Forearm	II°	Intern	Re- osteosynthesis			
F	13	Foot	III° b	Extern	Debridement	Soft tissue closure (flap)	Flap removal	5th Soft tissue closure (flap) 6th MR external fixator
M	16	Forearm	II°	Extern	MR external fixator, definitive osteosynthesis			
M	16	Lower leg	II°	Extern	Debridement	Soft tissue closure (flap)	MR external fixator	
M	11	Femur	I°	Extern	MR external fixator, definitive osteosynthesis			
F	11	Foot	III° a	Extern	Debridement	MR external fixator, debridement, soft tissue closure	MR k-wire, re-osteosynthesis	
M	16	Foot	II°	Extern	MR external fixator			
M	16	Foot	II°	Extern	MR external fixator			
M	14	Forearm	I°	Intern	Corrective osteosynthesis (six months later)	MR		
F	8	Lower leg	II°	Intern	MR			
M	12	Lower leg	II°	Intern	Fasciotomy	Debridement, secondary suture		
F	4	Foot	III° b	Extern	Debridement	Debridement	Debridement	5th to-16th : Debridement, sterile dressing change, then MR external fixator, definitive osteosythesis
M	14	Forearm	I°	Extern	MR external fixator, definitive osteosynthesis			
M	8	Lower leg	II°	Extern	MR external fixator, definitive osteosynthesis			

## Complications

The overall complication rate was 32.4%. Almost 80% of the complications occurred after injuries to the lower extremity. Detailed lists of complications according to the severity of the fracture can be found in Tables 4 and 5. We found a significant relationship between severity of the open fractures and occurrence of complications (*p* < .001). Furthermore, there was an accumulation of complications depending on the treatment method, with an increased incidence in relation to the expected values when using an external fixator (*p* < .001) and an increased incidence for non-primary wound closure (*p* < .001). A correlation between the occurrence of complications and the antibiotic treatment could not be shown (*p* = .179). With regard to the mechanism of injury, there was a significance with *p* = .007 and for the localization of the fracture with *p* = .008 respectively. In particular, there was an accumulation of complications in the context of a traffic accident and if the lower leg or foot is affected. In an analysis carried out individually for each severity grade, significant effects were only found in the III° open fracture patients for the treatment method using external fixator (*p* = .036) and traffic accident as mechanism of injury (*p* = .036). All statistical values can be found in Table 5. The infection rate was 4.1% (one osteomyelitis and two wound infections). The wound healing disorders of the former soft tissue defect were treated both operatively and non-operatively. Of the three injury associated nerve lesions two regressed completely. In an II° open fracture of the lower leg, a peroneal lesion was persistent. In an III° open femur fracture, growth plate involvement resulted in a shortening of the leg length and limited flexion in the knee joint due to soft tissue contracture. Eight of the ten compartment syndromes occurred after fractures of the lower leg (five primary and three secondary), two after Lisfranc fractures (both with primary onset). The re-fracture of the lower leg in an II° case occurred four months after completion of treatment due to a new impact. In another II° case, the distal phalanx D1 had to be amputated four weeks after the accident, in which a heavy weight was placed on the foot, following necrosis.

**Table 4:** Distribution of complications in relation to the severity of the fracture

Total *n*	Grade I° *n* (%)	Grade II° *n* (%)	Grade III° *n* (%)	Total *n* (%)
	33	25	16	74
Complications	4 (12.1)	10 (40.0)	10 (62.5)	24 (32.4)
Localization				
Humerus	-	1 (10.0)	-	1 (4.2)
Forearm	2 (50.0)	2 (20.0)	-	4 (16.7)
Femur	-	-	2 (20.0)	2 (8.3)
Lower leg	2 (50.0)	4 (40.0)	5 (50.0)	11 (45.8)
Foot	-	3 (30.0)	3 (30.0)	6 (25.0)
Type of Complications (incl. multiple combination)				
Compartment Syndrome	2 (6.1)	5 (20.0)	3 (18.8)	10 (13.5)
Primary	1 (3.0)	4 (16.0)	2 (12.5)	7 (9.5)
Secondary	1 (3.0)	1 (4.0)	1 (6.3)	3 (4.1)
Hardware failure	1 (3.0)	2 (8.0)	-	3 (4.1)
Nerve lesion	1 (3.0)	1 (4.0)	1 (6.3)	3 (4.1)
Secondary malalignment	1 (3.0)	-	-	1 (1.4)
Wound healing disorder	-	5 (20.0)	5 (31.3)	10 (13.5)
Refracture	-	1 (4.0)	-	1 (1.4)
Necrosis/amputation	-	1 (4.0)	-	1 (1.4)
Osteophytes	-	-	1 (6.3)	1 (1.4)
Osteomyelitis	-	-	1 (6.3)	1 (1.4)
Soft tissue contracture	-	-	2 (12.5)	2 (2.7)
Pseudarthrosis	-	-	1 (6.3)	1 (1.4)
Wound infection	-	1 (4.0) (MRSA)	1 (6.3) (Pseudomonas fluorescens, Enterobacter cloacae)	2 (2.7)

**Table 5:** Correlations between the occurrence of complications and various other factors for all patients and separated for severity of injury

		Pearsons chi-square or Fisher’s exact test *p*-values					
	*n*	Severity of injury	Treatment method	Wound closure	Antibiotic treatment	Mechanism of injury	Localization
Complications all patients	74	**< 0.001**	**< 0.001**	**< 0.001**	0.179	**0.007**	**0.008**
Complications Grade I	33	-	0.420	0.062	0.182	0.360	0.420
Complications Grade II	25		0.487	0.121	0.754	0.598	0.601
Complications Grade III	16		**0.036**	0.119	0.788	**0.036**	0.401

### Functional data

In 22 (29.7%) cases, the follow-up was less than three months, so 52 (70.3%) cases were included in the functional follow-up analysis. Functional data are shown in Table 6. The main reason for a follow-up of less than three months was the desire for further treatment close to home, other reasons were the completion of treatment within this time and in a few cases patients no longer attended their follow-up appointments and did not contact us again. Most cases (65.4%) were symptom-free at their last follow-up examination. Eight cases (15.4%) continued to have pain or were only pain-free with medication, half of which had an III° open fracture. After more than 12 months of follow-up, only one patient (1.9%) reported ongoing pain or the need for pain medication. In 15 (28.8%) cases, there was still a limitation of movement, although this was minor in most cases. Eleven (21.2%) of these 15 patients reported this within a 12-month follow-up period, while four (7.7%) reported this in a follow-up period of more than 24 months. In the case of the II° open fracture of the lower leg with a persistent peroneal lesion, a foot extensor paresis resulted. One (1.4%) case of an III° open Lisfranc fracture with residual pseudarthrosis resulted in significant degenerative changes of the entire Lisfranc line. In this case, however, pain was only present after long walking distances and problems with the fit of ready-made shoes were reported.

**Table 6:** Follow-up data of 52 cases with a minimum follow-up period of three months

Total *n*	Grade I° *n* (%)	Grade II° *n* (%)	Grade III° *n* (%)	Total *n* (%)
	22	17	13	52
Free of complaints	16 (72.7)	12 (70.6)	6 (46.2)	34 (65.4)
Pain	-	1 (5.9)	3 (23.1)	4 (7.7)
Pain medication	1 (4.5)	2 (11.8)	1 (7.7)	4 (7.7)
Restriction of mobility	6 (27.3)	5 (29.4)	4 (30.8)	15 (28.8)

## Discussion

Open fractures carry a potential risk of infection, complications and long-term consequences due to the bone communicating through the wound with the environment. As with adult management, there is general agreement on the need for initial treatment of open fractures in pediatric patients in the form of wound debridement, irrigation and initiation of intravenous antibiotic therapy. However, depending on the severity of the fracture, there is less agreement regarding fracture treatment, the timing of definitive treatment and the duration of antibiotic administration. To assess the complication rate in children and adolescents with open fracture in relation to grade of severity and other factors we performed a retrospective analysis.

I° open fractures are the most common type of open fracture seen in the pediatric population [6], which is reflected in our collective. There is no general consensus as to whether I° open fractures necessarily require surgical treatment. Iobst et al. reported infection in only one in 40 patients with non-operatively treatment consisting of irrigation and debridement in the emergency department and intravenous antibiotics as soon as possible [5]. Bazzi et al., in their retrospective review of I° open forearm and tibia fractures, also treated non-operatively, found no infections and only one delayed healing and consider this treatment to be safe [9]. In a very recent systematic review, Olatigbe and colleagues compared infection rates between non-operative management and surgical debridement in children with I° open upper limb fractures that did not require surgical fixation. Both groups showed comparably low infection rates of 0.3% and 0.4% [10]. The benefits of non-operative treatment include avoiding the risk of general anesthesia and minimizing costs by avoiding the surgery [11]. Critics of this method emphasize that most studies were underpowered and question their general applicability [1, 4]. However, all but one case with I° open fractures in our study were treated surgically. The complication rate in this group was 12.1% (four patients with five complications) with two compartment syndromes and each one hardware failure, nerve lesion and secondary malalignment. An increased risk of developing a compartment syndrome in high-velocity traumata, particularly in traffic accidents, and when the lower leg is affected is known from the literature [2, 12]. This was also true in these two cases. Infection rates for I° open fractures are given as 0–2% [2, 13, 14]. While there is a broad consensus on the prompt initial administration of antibiotics, the appropriate duration of therapy remains controversial [1, 7]. Depending on the treating physician and institution, the duration of antibiotic administration varies between approximately 24 and 72 h [3]. Dellinger et al. examined 248 adult patients with open fractures in a randomized, prospective double-blind study and compared a one-day with a five-day postoperative antibiotic treatment [15]. The result was that there were no significant differences in the infection rate for all three degrees of severity according to Gustilo and Anderson [15]. 90.9% of our I° open fracture cases received either single shot antibiosis only or single shot plus up to 10 days antibiosis. The infection rate in this group was 0%.

II° open fractures were the second most common in our collective. Infection rates from 1.9% up to 11% are reported for this group [14, 16], whereas our II° open fracture cases showed 4% infections (one wound infection) and 40% overall complication rate respectively. All five compartment syndromes (four primary, one secondary) in this group occurred after either a road traffic accident or a fall from a great height. The lower leg was affected three times and the Lisfranc joint twice, both of which, as mentioned previously, are already known to be associated with the occurrence of compartment syndromes [12]. One of these patients, whose tibial fracture was caused by a collision with a car while skateboarding, experienced multiple complications. He was diagnosed with an infection caused by a resistant organism, methicillin-resistant staphylococcus aureus (MRSA), suffered a perforation of the medial proximal prevot nail and, after treatment had already been completed, a new fracture of the tibia as a result of further trauma. With one affected patient, the amputation rate in our survey was 4% for II° open fractures and 1.4% for all patients included. In this patient, a large weight had fallen from a height onto the foot and several toes were crushed. Subsequently, phalangeal necrosis of the great toe developed, which ultimately led to distal phalanx D1 amputation. In their study, M’Bra et al. report a secondary amputation rate of 2.9% over a six-year period, including one patient after foot injury, however this was in an adult population [17].

As expected, with 62.5% the highest complication rate occurred in the III° open fractures. The duration of antibiotic treatment, especially for III° open fractures, remains controversial. Messner et al. state in their meta-analysis in adult populations of the existing evidence that the calculated pooled estimate of the infection rate of a longer course of administration of antibiotics (more than 72 h) did not differ significantly from a shorter course (less than 72 h) [18]. Even shorter courses of administration of antibiotics (24–48 h) were not different from treatment of more than 72 h [18]. The antibiotic therapy in our III° patients varied from single shot antibiosis only (three), antibiotics administered for up to seven days (two), antibiotics administered for more than seven days (four) and local antibiotic treatment (one). No differences were found in the occurrence of complications depending on the antibiotic treatment. The infection rate was 12.5% (one osteomyelitis and one wound infection) while literature data in this group vary between 8% in Skaggs and colleagues, 17.5% in Kelly et al. and up to 50% in Aulisa et al. [2, 14, 16]. Variability in infection rates may stem from differences in patient populations, injury severity and mechanisms, treatment protocols, classification approaches, and inconsistent definitions or reporting standards for infections. In 90% of the III° open fractures affected by complications, an external fixator was used for fracture stabilization. However, it is important to correctly differentiate between cause and effect. The reason for choosing an external fixator for osteosynthesis lies primarily in the severity of the injury, which in turn causes the complications described. From the literature, we know that external fixator is associated with various potential complications [2, 19]. According to Aulisa et al. the choice of fixation for open pediatrics fractures should be internal fixation whenever possible, referring to studies that have shown that this can be used up to III°A [2]. However, our patients with external fixation had three III° A, five III° B and one III° C fractures and sometimes, in severe cases, it is difficult to avoid external fixation [7].

The time between the injury and surgical treatment has also been subject of discussion in the past with the trend towards the earliest possible operation. Yet Skaggs and colleagues relativized the urgency of surgery in their study of 554 open grade I°, II° and III°A tibial fractures and found that an interval of more than 24 h had no effect on the rate of septic complications, provided early antibiotic prophylaxis was given [14]. 86.5% of our patients were operated within 6 h, a further 8.1% between 6 and 12 h so that we cannot make any valid statements about a delayed operation.

Our functional results suggest that children and adolescents typically demonstrate favorable long-term functional outcomes and minimal persistent pain following open fractures, which aligns with existing pediatric literature. Although open fractures in children are rare, they often have a better functional prognosis than in adults, provided timely and adequate treatment is given [1]. In particular, the low rate of chronic pain and associated functional limitations observed after more than 12 months is consistent with reports showing that children are able to minimize long-term consequences of open injuries due to their higher osteogenic capacity and vascularised periosteal structures [2].This study has limitations. The first lies in the nature of retrospective data collection relied on medical records, risking selection bias. Furthermore, the number of patients included is limited and due to a 1ost-to-follow-up rate of 29.7% long-term complication rates may be underreported. Subgroup analyses may be underpowered due to small subgroup sizes.

## Conclusions

In the present cohort, the overall complication rate increased markedly with the severity of the open fracture. This clear gradient highlights the importance of soft tissue damage as a significant factor in determining the outcome of open fractures in children and adolescents. However, it is essential to analyze the types of complications in more detail, as not all complications carry the same clinical significance or are specific to open fractures. Complications that are particularly feared in open fractures, namely wound infection and osteomyelitis, were relatively infrequent in the overall cohort. Osteomyelitis occurred exclusively in III° fractures and remained rare, while wound infections were documented in isolated cases with identified pathogens. By contrast, the majority of recorded complications were fracture-related, but not specific to open fractures. These included compartment syndrome, secondary malalignment, hardware failure, pseudarthrosis and soft tissue contractures. These complications were observed across all severity grades. Therefore, while higher-grade open fractures in children and adolescents are associated with a substantially increased overall complication burden, infection-related complications appear less dominant than traditionally feared.

## Data Availability

All data generated or analyzed during this study are included in this published article [and its supplementary information files].

## Abbreviations

SD: Standard deviation
ESIN: Elastic stable intramedullary nailing
MRSA: Methicillin-resistant staphylococcus aureus
SS: Single shot
